# A Collection of Memoirs on Military Medicine, Surgery, and Pharmacy

**Published:** 1846-07

**Authors:** 


### Recueil de Memoires de Medicine de Chirurgie et de Pharmacie Militaires. Vol. 59. Paris, 1845. 8vo. pp. 415.

### A Collection of Memoirs on Military Medicine, Surgery, and Pharmacy.

The present volume of the "Recueil" contains several interesting papers, an analysis of some of which we proceed to lay before our readers.

### I. On the Treatment of Sprains. By Dr. *Poullain* of the Military Hospital at Lyon.

Sprain is an accident of very frequent occurrence among soldiers, especially those of them who belong to the cavalry. During the seven or eight years that Dr. Poullain has been attached to the 1st Regiment of Dragoons, he has had from 70 to 80 more or less serious cases under his care. The object of this paper is to detail the plan of treatment followed in these and other cases, which has been so successful that it has been very rarely found necessary to send the men into the hospital. The usual mode of treatment by the application of leeches and emollient cataplasms is not

---

only inefficacious, but even hurtful; for the hospital to which Dr. P. is attached being that to which the soldiers are sent for the benefit of the mineral waters (Eaux de Bagnoles), he is in a condition to prove that, of every hundred patients sent there, from 25 to 30 are so on account of chronic articular swellings, consecutive to this mode of treating sprains, several of which eventually require amputation. He possesses notes of more than 103 such cases, exhibiting the uselessness or danger of the practice, a few of which are inserted in the present paper. Larrey and other celebrated surgeons have likewise protested against this mode of treatment. Professor Begin observes, in his Elements of Surgery:—

"Some persons having an exaggerated fear of inflammation, combat it in sprain by means of warm topical emollients, repeated local bleedings, and frequently-renewed applications. This plan seems to me defective. It does not allow the parts to remain absolutely at rest, it favours their tumefaction, *engorgement* and suppuration, while the recoveries it procures are tedious, purchased with risk, and less complete than those which follow the use of repercussives."

M. Baudens, senior surgeon of the military hospital of Val-de-Grace, is likewise of precisely the same opinion.

"Leeches applied immediately after a sprain," he says, "induce an afflux of blood to the part which we should do all in our power to prevent. Cataplasms maintain this fluxion, and after the foot has been macerated 10 or 15 days in such an application, it remains pasty and deprived of its elasticity, and, when not rendered liable to a degenerescence, which not unfrequently leads to the necessity of amputation, the difficulty of walking and persisting *engorgement* at least manifest its debility."

The means which Dr. Poullain and the above authorities recommend in lieu of leeching and cataplasms is the *immediate and continued application of cold* by immersing the part in water. The cure is not only prompt but complete, inasmuch as there is no remaining *engorgement* to lay the foundation for future mischief, and the patient is enabled to employ the joint as actively as heretofore. This would be a great point gained even if the time consumed in the treatment were as great in the one plan as the other, which it is not. Many cases of its success are related in the paper, and although, of course, in the great majority of instances, the ankle is the joint affected, sprains of other joints may be treated in just the same manner, except that in those, such as the knee, in which immersion may be difficult, the application of wet compresses or irrigation may be substituted. The treatment indeed is not novel, for it was recommended by Boyer, and more recently by M. Begin.

"Of 90 patients whom I have treated by the aid of cold and resolvents, 23 were cured in 6 days, 10 in 8 days, 22 in 11 days, 28 in from 11 to 15 days, 4 in from 20 to 25 days, and 3 at the end of a month. None of these persons have continued lame. Seven felt the effects of their accident for several months, without, however, being prevented attending to their duties, and becoming quite cured. * * * * * * If this mode of treatment has incurred blame at the hands of some surgeons, it is because it has not been sufficiently promptly and freely employed, and it is therefore necessary to lay down some rules upon this point.

"The immersion should be resorted to as soon after the accident as possible. Recourse may be had to it also three, four, five, six, or even 12 hours after, but then its sedative effect is less prompt and the cure more tedious. The foot should remain at least *two hours* in the bath, and oftentimes much longer. It may sometimes be left in for entire days; and as a general rule the part should not be removed until it becomes completely cooled, the water being renewed as often as it becomes warm. This prolongation is easily obtained, for, after the first hour or so (during which the pain is sometimes almost insupportable,) the immersion becomes bearable, and the patient is himself very desirous for its continuance. Iced water does not possess any superior efficacy to that of a temperature of 37° or 39°, provided this be equally maintained. As soon as the limb is removed from the bath it must be surrounded by a roller previously moistened with Goulard water or camphorated spirit, some of which must afterwards be applied to it from time to time. So effectually are congestion and swelling in this way diminished, that the bandage usually becomes loose within the 24 hours. It must be re-applied until all swelling and pain has disappeared, which is generally the case in from three to six days. The patient may now be allowed to walk, continuing however the use of a bandage for ten or twelve days.

" If 14, or even six or twelve, hours after the application of the wet bandage, pain still continues, or throbbing is felt by the patient, it must be taken off, and the limb again immersed in the water for a longer period than at first, even for a whole day if requisite. This second immersion is sometimes unsuccessful, but fortunately it is very rarely required, as the first almost always suffices.

" If the sprain is several days old, the limb swollen and painful while nothing has been done for it, a free local bleeding is a necessary preliminary, after which the bandage and cold lotions or perhaps immersion itself, should at once be resorted to. These means are, however, now of far less service than when employed soon after the occurrence of the accident."

When the sprain has been badly treated the joint may become the seat of a chronic enlargement, which is dissipated with difficulty, and only after the persevering use of compression. MM. Begin and Velpeau, indeed, employ this in the earliest stage of sprain as a powerful means of preventing inflammatory swelling. Dr. Poullain employs to this end a starched many-tailed bandage. Whatever means are used the case is tedious, and may also require the aid of stimulating liniments, or, if very obstinate, of the douche as employed at the mineral springs, and even this does not always dissipate the enlargement.

The Editors of the " Recueil," in a note to this communication, corroborate the statement of the efficacy of the treatment of sprain by immersion, provided this be uninterruptedly continued. They state, also, that M. Baudens of the Val-de-Grace, so far from finding the first period of the immersion to be attended with pain as related by Dr. Poullain, has always observed marked relief from this symptom to occur at once. This practitioner employs this means with great success, not only in sprains, but also in every case of acute traumatic inflammation. Several cases are cited of the beneficial results following the continuous application of ice, cold lotions, &c., too, in cases of bad compound fracture, wounds of the joints, &c. &c.; general bleeding, however, being also generally resorted to in serious cases. In the treatment of sprain, M. Baudens has usually had to prolong the immersion for a longer period than Dr. B. has found requisite. He observes it occasionally happens that, during the immersion, the pain which had subsided is renewed with great severity. This is a sign of threatened congelation of the part, which must be removed from the bath, bathed in tepid poppy-water, and rolled with a starch bandage.

### II. On the Treatment of Orchitis. By *M. Songy*, Assistant Surgeon 3rd Chasseurs in Africa.

M. Songy observes, practitioners have long recognized that, however distinctly inflammatory the affection of the testis may be at its origin, its results soon become modified by the nature of the tissues, and that, although sometimes the disease may be a pure inflammation of the terticular parenchyma, at others, it consists of an œdematous condition of the external coverings, or of an effusion into the tunica vaginalis. This view of the case led to modifications in treatment, and compression employed by Fricke of Hamburg, was also adopted by Velpeau and Ricord, and recommended by them as a speedy means of cure. Nevertheless, the majority of practitioners have continued to employ antiphlogistics for the relief of the early symptoms, and resolvents to dissipate the consecutive indurations, which are often-times so obstinate, and frequently form the germs of future attacks. Even supposing these means were more efficacious than they really are, the instances are numerous in both civil and military practice, in which their employment, in such an affection, is attended with inconvenience or annoyance.

"I do not say that any means which has been hitherto found useful should be abandoned, for each may have its value. My object is to introduce a mode of treatment which I believe has not been yet employed, and which I regard as very successful as well as easy of application. This new agent, which has never failed me during the four years I have employed it in our military hospitals, is *cotton*, or, where I have not been able to obtain this, as in some districts of Africa, sheep's wool. * * * * * * * I consider that, in orchitis, we may have either an inflammation of the testis itself in which the coverings of the gland may participate, or a phlegmasia whether of the epididymis or the tunica vaginalis, with œdema of the scrotum and more or less extension to the gland; thus, distinguishing these, as does M. Vidal de Cassis, into *parenchymatous orchitis* or inflammation of the substance of the testis, and *membranous orchitis* embracing the varieties known by authors as vaginitis and epididymitis. Is this division of practical importance? I cannot doubt it, and authors have tacitly admitted it to be so. They have described orchitis which required little else than hygienic treatment, viz. the membranous form; but they have also described orchitis with violent pain and symptomatic effects, such as hiccough, vomiting, convulsions, fever, &c. which all agree arise from the propagation of the inflammation to the substance of the gland, requiring the most active treatment. These cases are exceptional, and we have only to enquire whether antiphlogistics are requisite for the cure of the membranous form which constitutes the great majority of cases, or whether mere hygienic precautions will not suffice. With few exceptions physicians and surgeons at all periods have prescribed *absolute rest of the organ affected* as an indispensable condition of cure. But we must not confound this with the absolute *rest of the body*, by keeping in bed, which patients are ordered to observe at the commencement of the affection, for they are two different things."

M. Songy then points out the inefficiency of the means usually employed to secure this absolute rest of the organ, viz. the constant application of cataplasms or the strapping up the testis. As soon as the cataplasm is cold and dry, it may become a source of irritation to the part, and, ceasing to be accurately applied to the surface of the organ, it admits of injurious motion and friction. These objections do not apply to the same extent to strapping the testis. But first the preliminary removal of the hairs of the scrotum is a painful and difficult operation, and their reproduction will yet after a while necessitate the re-application of the plaister, which cannot be removed without exciting an injurious traction. The constriction of the cord by the plaister also may give rise to much pain; while, when the tumour has diminished somewhat, and before the plaister is renewed, an injurious mobility is allowed to the testis, described by the patients as "the nut rattling in its shell."

"It may be objected to me that I insist too much upon the necessity of the absolute repose of the organ in an affection generally regarded as a slight one; but, in external as in internal pathology, nothing should be neglected. To the mildest symptoms sometimes have succeeded diseases almost beyond the reach of art. And, then, is chronic orchitis so insignificant an affection, that the practitioner can venture to neglect considerations mistakingly represented as too minute? Will, then, cotton or wool more effectually maintain the organ in a state of absolute rest, without preventing the patient following his ordinary occupations? Once applied, as it undergoes no change in position, it need never be renewed, and even the diminution of the tumour does not exact this, inasmuch as, by its natural suppleness and elasticity, the cotton always as it were moulds itself around the parts which it envelops. Moreover, these same properties, by deadening any inconsiderate movements of the patients, prevent the effects of pressure or friction of the parts so injurious in an affection in which the local sensibility is so exalted that the slightest touch induces excessive pain. When placed in contact with the scrotum, the cotton at once produces a sense of comfort soon followed by a mild and moist warmth, the cutaneous transpiration being sensibly increased. * * * * * * *
But to obtain the advantageous results which cotton has furnished us with for several years some precautions are indispensable in its application.

"After having chosen a suspender which is perfectly adapted to the parts it is destined to sustain, and fastened it around the body, the surgeon surrounds the scrotum over its whole extent from the perineum to the root of the penis with a layer of carded cotton (or of thick wool so prepared as to present the tomentous aspect of cotton) of about an inch in thickness. It is then to be placed in the suspender, but at this period, if the success of the case is to be secured, the practitioner must gently raise the tumour in such a manner that the testis may become applied as near as possible to the ring, in which position it is to be maintained. Thus placed, the tumour, which from vertical has become horizontal, will not exert any influence upon the *engorgement* by its weight, and the testis, thus bolstered up, is scarcely brought into the horizontal position before the pain diminishes, and ere long disappears."

Some cases are added exemplifying the rapidity with which relief from suffering is obtained, and the ease with which patients so treated can avoid the inconveniences of confinement.

### III. On the Use of the Suture in Wounds of the Neck. By Professor *Bertherand*, of the Strasbourg Military Hospital.

Most surgeons have objected to the use of the suture except in superficial wounds of the neck, and Dieffenbach rejects it altogether. He observes that, owing to the loose and extensible condition of the subcutaneous cellular tissue of this part, and the consequent facility with which the skin glides over the subjacent layers, it results that any approximation of the edges of the wound is but superficial, the deeper-seated parts remaining separated. Between these, cloaca are established, pus accumulates and degenerates, and more or less extensive burrowing and other accidents ultimately result. But it is to be observed, that the suture is not the cause of these, but the deceptive security of the superficial union of the parts. A better-founded objection to the suture is that, when it only implicates the skin, the mobility of this, already adverted to, may lead to irritation and ulceration of the apertures, and a clean section be thus converted into an irregular wound of tedious cicatrization.

During the author's attendance upon the *ambulances* in Africa he had frequent occasion to observe the insufficiency of these superficial re-unions, and of the various contrivances put into force to keep the parts in contact. It seemed to him possible, by passing the suture through the deeper-seated parts, to substitute for this great mobility of the skin so incompatible with firm adhesion, very limited movements of the entire parts in their relative positions. A case soon offered itself for putting the idea into practice. A soldier was brought to the *ambulance*, having a wound on the right side of the neck, eight or ten fingers' breadth in length. The sterno-cleido-mastoideus was divided through, but the carotid had escaped, and was observed pulsating in the anterior angle of the wound. Behind, the trapezius was transversely divided to the extent of about an inch and a half. Several large coagula were washed out of the wound, and two small branches of the superior thyroid submitted to torsion. A long curved needle, carrying a double thread, was passed through as great a thickness of the muscular substance as possible, and its point brought out about an inch from the edge of the wound. A quilled suture, penetrating thus deeply, was formed at each extremity of the wound, and two others, more superficial, brought the intermediate parts into contact, Adhesive strips were placed between these, and the whole covered with a "solid dressing." The head was kept bent to the right side, and the greatest quietude enjoined. Three of the sutures were removed on the 6th day, and the remaining one on the 9th. Adhesion went on satisfactorily; and, on the 20th day, solid food was given the patient, he being considered perfectly cured.

Another case is related which, however, did not come under Dr. Bertherand's care until the sutures had already been inserted. A soldier attempted to cut his throat, and inflicted a large incision upon it, a small fragment of the thyroid cartilage being observed to be floating in the wound. Several coagula filled the wound (which had been already strapped up), and prevented the issue of air by the aperture. The voice was scarcely affected. The edges were united by two sutures and dressed. The patient was bled, and again in the evening, as he was seized with violent delirium. Afterwards he became calm and dejected, and determined to abstain from all food whatever, and would reply to no questions. Dr. B. regarded his self-imposed immoveability of parts as highly useful, especially as his former violence had not disturbed the adhesive process. At the end of a week the sutures were removed, and, as two days afterwards the patient still continued obstinate, Dr. B. said aloud, near his bed, that as he was quite well he should order his dismissal, at the same time that he prescribed for him a better diet. He yielded to this, took food, recovered his strength gradually, and was dismissed the 20th day after the accident—fasting for *eleven days* having been thus borne by one whose digestive organs possessed all their activity.

Dr. Bertherand does not mean to state that sutures are applicable to every wound of the neck. As a general rule, the regularity of the section is of more consequence than the superficiality of the wound. Irregular wounds, with loss of substance, excavations, rough walls, or communicating with the œsophagus, do not admit of the suture. The editor of the "Recueil" observes, that M. Begin employs sutures in some forms of wounds of the throat, and that the necessity of embracing a great thickness of the lips of the wound in the ligatures has been long acknowledged by M. Baudens in his treatment of the wounds which result from the extirpation of cervical ganglions. As a general rule, he has found wounds so treated unite by the first intention, and the patients have been cured in from 8 to 10 days.

### IV. Report of several Cases of Cerebro-spinal Meningitis which occurred at Douéra (Algeria) in 1845. By Dr. *Magail.*

Cerebro-spinal meningitis prevailed epidemically in various garrisons of France from 1837 to 1842; and the Report of Dr. Magail exhibits the disease identical in all its characters, but far less extensive in its prevalence, in Africa. Between the 4th and 17th of February, 10 soldiers were attacked, when the epidemic ceased, to re-appear again on the 21st March, between which and the 26th April twelve new cases occurred. The subjects of the first epidemic were robust, healthy men, of from 21 to 30 years of age, all of whom had been in the service for one or more years. Four of them were drunkards, two nostalgic, and one very low-spirited. In explaining various epidemics, much stress has usually been laid upon the bad lodgement and over-crowding of troops, their excessive fatigue, the bad condition of the barracks, and the dissipated conduct of the soldiers. All such causes should rather operate upon the feeble and debilitated than upon the robust, while the condition of the soldiers at Douéra, in respect to air, dress, food, and exercise, is excellent. The invasion of the disease was always sudden, coming on in cold, damp weather, with violent cerebro-spinal pain, delirium, and other symptoms of excitement, and passing rapidly into a state of collapse. The pulse was however small and soft, and ranged from 106 to 114; vomiting and obstinate constipation were always present, while the urine was normal as to quantity, but gave out a very distinct odour of "bat's." The penis was sometimes erect, some patients presented a more or less complete trismus, and others subsultus. Every patient laid exclusively upon his side, usually the left, with the extremities bent up, and the head drawn too much forward or backward, the muscles of the neck and extremities being more or less rigid. Blood drawn from a vein was always found destitute of buffiness, and possessing but a small proportion of serum. The collapse came on from 12 to 48 hours. The prognosis was very unfavorable, recovery never occurring after the period of collapse had commenced. Of the ten patients six died in from 8 to 23 hours after the collapse had commenced—this generally occupying a third of the whole duration of the disease. Some of those patients died within 21 and 23 hours, and the longest lived only 72 hours after the commencement of the attack, and yet, in all, distinct *suppuration* was discovered. The dura mater, sinuses, and veins of the surface of the brain were found gorged with blood; but the pus presented great varieties as to its situation, consistency, and quantity, the base of the brain being the spot it was oftenest found at. It not only existed at various other parts of the exterior of the brain, but also in the cellular tissue connecting the arachnoid and pia-mater. The ventricles were filled with a lactescent or sanguinolent serosity. Pus was found in the spine only in three subjects, and in these resembled a band covering the posterior portion of the narrow from its origin to the cauda equina. The spinal marrow was however often found softened, even when the condition of the substance of the brain in this respect was normal, which it usually was.

The *treatment* of the disease, to be of any avail, must be vigorously active. General bleeding to syncope is necessary, although for the production of this sometimes the loss of from 40 to 120 ozs. was required. While the blood was flowing from the arm, hundreds of leeches were placed upon the head, neck, spine, and anus, and cupping-glasses applied to various parts. Hot water was applied to the legs and thighs, cold compresses to the head, and active enemata administered. Shortly after reaction was endeavoured to be produced by active friction and sinapisms applied to all the extremities. When this seemed of no avail, the actual cautery was applied at various points along the spine and blisters inside the thighs. Quinine was tried with no benefit, and tartar-emetic with some apparently good effect. Croton oil, in doses of three or four drops during the 24 hours, seemed also of some advantage, when it induced copious stools.

The *second* epidemic was somewhat less violent, and only attacked young soldiers who had just joined the regiment, the invasion of the disease taking place, as in the other, constantly at night. In these cases general bleeding to syncope was resorted to as early as possible, and a nasal hæmorrhage maintained by leeches applied to the nares. This latter was continued as long as the symptoms of excitement lasted, *i, e.* during 48 or 64 hours; free purging by croton oil being also advantageously maintained during the same period. Sinapisms were applied to the extremities, and embrocations to the spinal column. In the present epidemic, however, M. Magail abstained from making any active application, such as leeches, blisters, moxas, cautery, &c. to the spine itself, "experience having too strongly proved to me that to act in this manner on the spinal column is only to augment the mischief by favouring suppuration, and thus leading to a fatal termination." Ten out of the twelve cases of this epidemic were cured, although in eight of them symptoms sufficiently alarming had manifested themselves. The patients who died did not apply for treatment until the disease had much advanced.

M. Guyon, in a note to this communication, observes that a similar epidemic was observed at Douéra, in 1840, when of 13 soldiers attacked only one survived. Other cases have been observed in other parts of Algeria, but he is unacquainted with the particulars of these.

### V. On Phthisis among Soldiers. By M. *Godelier*, Professor of Internal Pathology at Strasburg.

This paper, for which its author obtained a gold medal in 1844, was prepared in answer to the following question, proposed for discussion by the Council of Health for the Army—"*What are the causes of the frequent development of phthisis among soldiers, and the most efficacious means of preventing and treating this disease?*"

M. Godelier first endeavours to ascertain in what proportion the deaths of phthisis occur in military life compared with those occurring in civil life: but he soon finds himself at a loss from the non-existence of any statistical documents which can elucidate the point. He is indeed obliged to have recourse to the Registrar's and Army Reports of this country, and assume that a like proportion prevails in France. We have so continually had dinned into our ears the excellence of the continental arrangements for internal administration and the collection of facts, that it is not unpleasing to find our superiority in this respect cheerfully acknowledged by every writer who has occasion to search into these matters for himself. It is true that, in some of the more despotic states, much information is accumulated in the various *bureaux*, but only for the use of the government authorities themselves, and even in enlightened France itself there is a great backwardness in publishing many documents which yet might prove very useful. The Reports of the Registrar-General and Poor-Law Commissioners, and those of Messrs. Tulloch, Wilson, and Chadwick, diffused far and wide, are spreading useful information among all nations, at the same time that they do honour to that one whence they emanate. We need not follow M. Godelier through his calculation, the conclusions to be drawn from which he acknowledges to be only approximative. He says:

"From an attentive examination of these documents it results—1. That of 10,000 soldiers, about from 50 to 60 die of phthisis. 2. This figure being somewhat lower than that which attaches to the males of the civil population of the same age (between 20 and 30), leads to the presumption that the soldier is less exposed to pulmonary tubercularization than these. 3. The difference being but slight, and the data furnishing it not presenting all the certainty desirable, it is allowable to suppose that it might be altered by an examination of additional facts. 4. It would be unlogical to assume a doubtful result, and search at present for the causes which render the soldier less liable to phthisis than the civilian, a state of things not yet sufficiently demonstrated, and one which, if true, may be explained by the fact of a soldier being a picked man from the mass of the population."

We have adverted, in our article upon Dr. Thurnam's "Statistics of Insanity," in the present Number, to some *statistical errors* which have obtained currency, and M. Godelier signalizes others. Speaking of an article on the Mortality of the French Infantry in Vol. X. of the *Annales d'Hygiene*, he observes,—

"This is the only work in France, relating to the army, where the amount of deaths is compared with that of the effective force. Such a condition is indispensible for the establishment of any true conclusion, and yet it is so generally neglected, that I cannot forbear pointing out some of the errors which result. It is customary to be contented with comparing in statistical tables the amount of deaths furnished by a disease with the total number of persons attacked by it, or with the entire mortality. Thus, of 100 cases of pneumonia 5 died; in 100 deaths, 20 arose from phthisis. The first of these comparisons may serve to shew the mortality of a given affection under particular circumstances. The second professes to indicate the part which a disease plays in the general mortality, or sometimes the *frequency of the disease.* I maintain that it is a grave error to take the relation which deaths from phthisis bears to the other deaths as a measure of its frequency; for the total of deaths being incessantly variable, its elevation or descent may change in a contrary direction the value of the relation, although the number of deaths from phthisis continue the same.

"Suppose, *e. g*, three corps, in the 1st of which the proportion of the mortality from phthisis to the general mortality is $\frac{1}{10}$, in the 2nd $\frac{1}{20}$, and in the 3rd $\frac{1}{30}$, it would be concluded that phthisis had committed by far the greatest ravages on the first of these, while nothing would be less certain than that it had; for, if the effective force of each corps had been the same, and each had lost the same number of consumptive soldiers, their equality in this point is obvious—the difference consisting only in the augmentation of the number of the other deaths. Thus:

| | Effective Force. | Total Deaths. | Deaths from Phthisis. | Proportion. |
|---|---|---|---|---|
| 1st | Corps of 20,000 | 100 | 10 | $\frac{1}{10}$ |
| 2nd | Corps of 20,000 | 200 | 10 | $\frac{1}{20}$ |
| 3rd | Corps of 20,000 | 300 | 10 | $\frac{1}{30}$ |

"This is, however, the error which disfigures and renders sterile the majority of this kind of statistical documents. It has diminished the value of the labours of Benoiston de Chateauneuf, and Lombard of Geneva, upon the influence which professions exert in the production of phthisis. They have accurately counted the number of deaths which it had caused in each of these; but since they were unable to compare them with the unknown total of individuals employed in each profession, how could they draw conclusions as to the relative frequency of the disease? Thus, when M. Benoiston de Chateauneuf confines himself to stating that, of 17,209 deaths in the French infantry, 1,260 were due to phthisis ($\frac{1}{14}$ or 0.074), the information is of little importance: but if I learn that these 1,260 deaths took place in 801,748 soldiers ($\frac{1}{636}$ or 0.0016), I acquire by this fixed term of comparison a relation of great value, which is of service as an approximate measure. Statistic results, from whatever authority they emanate, require to be controlled by new results before they can obtain the force of laws. All the sagacity of the statistician is often powerless for the correction of the defective elements of his calculations. New series of facts are requisite to palliate or correct his errors."

M. Godelier enters into a long examination of the various circumstances in the condition of the soldier which may predispose him to the disease, and thus sums up—1. A residence in Africa appears to be favorable to European troops, while one in the West Indies is quite the reverse. 2. The influence of birth-place is not ascertained. 3. The officer is much less liable to phthisis than the private, and he owes his comparative immunity much more to the influence of hygiene than to that of age. 4. Those branches of the service are least exposed to the disease in which are the strongest constituted men, and which adopt the most regular system of exercises, and the best hygiene. 5. All things being equal, the constancy, regularity, and variety of exercises contribute most to exemption.

6. Bad conditions of the air, food, lodging, and exercise exert an acknowledged influence in producing phthisis, and as these conditions are in some measure inherent to the soldier's life, they must exert the same effect upon him. And thus the vitiation of the air in his crowded sleeping-rooms, the too great uniformity of his diet, the damp and coldness of some barracks, and insufficient or injurious exercises, all tend to this end. 7. The existence of chronic disease, necessitating a long sojourn in the hospital, predisposes to phthisis. 8. The mode of life the soldier leads exposes him to numerous occasional causes which may act on the predisposed.

The chapter on *prophylaxis* enforces the necessity of the proceedings which the above conclusions obviously indicate; and M. G. also suggests the propriety of allowing valetudinarians an opportunity of re-enforcing their health by a visit to Algeria, the climate of which seems so adverse to the production of tubercle. In regard to the *treatment*, the author speaks of the existence of the power of detecting the early stages of the disease by a combined observation of the physical, local, and general signs, with a positiveness which, unfortunately, facts do not warrant; and he also promises a success from the adoption of general hygienic precautions and local antiphlogistics, in a far more sanguine manner than we should have expected in so cautious an observer. However, this little avails the soldier, since M. Godelier admits that early or anticipatory treatment is incompatible with the exigencies of the service; and recommends that whenever incipient phthisis is detected, he may be allowed his discharge.

